# Strategies for Vaccination: Conventional Vaccine Approaches Versus New-Generation Strategies in Combination with Adjuvants

**DOI:** 10.3390/pharmaceutics13020140

**Published:** 2021-01-22

**Authors:** Abdellatif Bouazzaoui, Ahmed A. H. Abdellatif, Faisal A. Al-Allaf, Neda M. Bogari, Saied Al-Dehlawi, Sameer H. Qari

**Affiliations:** 1 Department of Medical Genetics, Faculty of Medicine, Umm Al-Qura University, P.O. Box 715, Makkah 21955, Saudi Arabia; faallaf@uqu.edu.sa (F.A.A.-A.); nmbogari@uqu.edu.sa (N.M.B.); 2 Science and Technology Unit, Umm Al Qura University, P.O. Box 715, Makkah 21955, Saudi Arabia; 3 Department of Pharmaceutics, College of Pharmacy, Qassim University, Qassim 51452, Saudi Arabia; a.abdellatif@qu.edu.sa; 4 Department of Pharmaceutics and Industrial Pharmacy, Faculty of Pharmacy, Al-Azhar University, Assiut 71524, Egypt; 5 Department of Laboratory and Blood Bank, Molecular Diagnostics Unit, King Abdullah Medical City, Makkah 21955, Saudi Arabia; 6 Regional Laboratory, Makkah 25321, Saudi Arabia; sdehlawi@moh.gov.sa; 7 Biology Department, Aljumum University College, Umm Al-Qura University, Makkah 21955, Saudi Arabia; shqari@uqu.edu.sa

**Keywords:** vaccine adjuvant, viral vector, DNA vaccine, RNA vaccine, nanoparticle

## Abstract

The current COVID-19 pandemic, caused by severe acute respiratory syndrome-coronavirus 2 (SARS-CoV-2), has raised significant economic, social, and psychological concerns. The rapid spread of the virus, coupled with the absence of vaccines and antiviral treatments for SARS-CoV-2, has galvanized a major global endeavor to develop effective vaccines. Within a matter of just a few months of the initial outbreak, research teams worldwide, adopting a range of different strategies, embarked on a quest to develop effective vaccine that could be effectively used to suppress this virulent pathogen. In this review, we describe conventional approaches to vaccine development, including strategies employing proteins, peptides, and attenuated or inactivated pathogens in combination with adjuvants (including genetic adjuvants). We also present details of the novel strategies that were adopted by different research groups to successfully transfer recombinantly expressed antigens while using viral vectors (adenoviral and retroviral) and non-viral delivery systems, and how recently developed methods have been applied in order to produce vaccines that are based on mRNA, self-amplifying RNA (saRNA), and trans-amplifying RNA (taRNA). Moreover, we discuss the methods that are being used to enhance mRNA stability and protein production, the advantages and disadvantages of different methods, and the challenges that are encountered during the development of effective vaccines.

## 1. Introduction

The outbreak of the COVID-19 pandemic [1,2,3,4], which was caused by severe acute respiratory syndrome-coronavirus 2 (SARS-CoV-2), has triggered a global race to develop effective vaccines. Approximately 150 different research groups are currently involved, and more than 100 clinical trials have been initiated since the outbreak was first reported [5]. They all have the singular goal of developing and producing an antiviral vaccine that is effective in individuals of all age groups with all conditions, and, thereby, control the course of the pandemic. Nevertheless, the development of a vaccine is a laborious process, the mass production, distribution, and administration of which present extraordinary challenges, particularly in developing countries. Accordingly, the strategy that has been employed for vaccine production needs not only to take into consideration the effect of the vaccine on the immune system and its efficacy against the virus, but also the procedures for mass production, distribution, storage, and mass vaccination [5]. To this end, the participating research groups are employing a diverse range of different formulations, techniques, and strategies to produce effective vaccines against SARS-CoV-2. In this regard, there are four main methods of vaccine development, namely, employing pathogens (inactivated or with low virulence) for the production of vaccines; recombinant protein vaccines; vector-based vaccines that include DNA vectors or viral vectors; and, the latest technology using RNA molecules for vaccination. Among these, the more innovatory next-generation vaccines only use a part of the virus protein structure and, thus, can be expected to have a superior safety profile. However, these novel vaccines tend to have low immunogenicity and they often fail to induce a sufficient immune response. Consequently, we also describe the use different adjuvants, which can be employed in order to enhance immunogenicity and establish an enduring immune memory.

## 2. Traditional Vaccines

Historically, the first vaccines were based on pathogens with reduced virulence. Among the pioneers in this field, scientists, such as Plett and Jenner, used the cowpox or horsepox [6,7,8] virus in order to generate vaccines against smallpox. These types of vaccine with relatively low virulence have several advantages, notably only causing mild infection with symptoms that are similar to those of the target pathogen, and the body subsequently develops a strong immune response, with immunity potentially persisting for years. However, such traditional vaccines have one particularly vital drawback, namely, a high infection risk due to the potential for pathogens with low virulence to become more virulent [9]. The second method of traditional vaccination involves the administration of inactivated vaccines, which are safer than the first type. However, the use of such vaccines necessitates multiple injections in order to achieve strong and long-lasting immunity. Although live attenuated or inactivated vaccines can be more readily and rapidly developed than other vaccine types [10], these traditional vaccines tend to have a poor safety record, and a defect in the production process can potentially be a source of disease outbreaks. Indeed, such an incident occurred in the year 1955, when the administration of a defective polio vaccine caused 10 deaths, paralysis in 200 recipients, and a total of 40,000 cases of polio infection [11]. Accordingly, the development of alternative vaccines with better safety profiles is a priority.

## 3. Next-Generation Vaccines

In 1990, Wolf et al. demonstrated that mice injected with plasmids harboring a cloned protein subsequently showed an expression of the transgenic protein cloned in the plasmid DNA [12]. These observations provided an impetus for the development of a new strategy of vaccination, and marked the advent of an era of next-generation vaccines. The initial strategy adopted for these novel vaccines was a DNA-based technique, which was subsequently followed by the development of viral vectors, including adeno-associated virus (AAV), lentiviral, or adenoviral vectors for vaccination, and, more recently, by RNA-based vaccines. The salient point of this research is that it demonstrates that only a portion of the viral protein structure is sufficient for promoting immunity against a given pathogen. Consequently, these innovatory vaccines tend to only include a specific viral antigen, instead of employing the entire pathogen, thereby resulting in a better safety profile [13]. However, the design of such vaccines requires a more in-depth understanding of viral structures and the interaction between viral proteins and host cell receptors, and, accordingly, these next-generation vaccines tend to require a lengthy phase of preliminary studies before development can commence.

### 3.1. Recombinant Protein Vaccines

Recombinant protein vaccines are based on the use of recombinant viral structural proteins to induce an immune response. In this respect, the SARS-CoV-2 genome comprises four structural proteins, namely, membrane, envelope, nucleocapsid, and spike proteins. Among these, the spike protein is of particular importance, given that it interacts with angiotensin-converting enzyme 2 (ACE2) receptors that are localized on the surface of host cells, thereby facilitating endocytosis [14]. Consequently, most vaccination strategies for the SARS-CoV-2 virus have focused on this protein, owing to its importance in the virus lifecycle. However, vaccination with whole spike protein has been shown to promote liver damage in treated animals [15] and, thus, the use of only a part of this protein, such as the receptor-binding domain (RBD), which interacts with the ACE2 receptor protein, is considered to be the best alternative with respect to producing a safer vaccine [16]. Initial research in this regard suggests that immunization with recombinant protein or only the RBD results in the production of neutralizing antibodies [16,17,18]. Observations indicated that the protein is processed by dendritic cells, followed by the presentation of the antigen to naïve B and T cells, resulting in their activation and subsequent immunity development. However, the use of this strategy for immunization has a notably important drawback, namely, that, owing to the use of only a small part of the protein for immunization, specific immune reactions induced by the vaccine confer only partial protection [18,19,20]. Moreover, these immune reactions tend not to be particularly strong [21]. Consequently, vaccination with recombinant proteins necessitates the use of substrates, referred to as adjuvants, to boost the immune response. The use of such adjuvants enhances antigen presentation in antigen-presenting cells (APCs), thereby enhancing vaccine efficacy and resulting in long-term protection.

### 3.2. Plasmid DNA Vaccines

Wolff et al. demonstrated that intramuscular injection of nucleic acids resulted in the in vivo expression of a protein encoded by plasmid DNA [12], and it was later shown that vaccination with plasmid DNA can induce a strong immune response, as mentioned previously [22,23,24]. Collectively, the findings of these studies have provided evidence of the potential of plasmid DNA to produce immunization on injection. Subsequently, researchers began to examine the utility of DNA vaccines for the treatment of cancer, infections, and autoimmune diseases, including allergies [25]. However, the early-stage clinical studies in humans tended to be unsuccessful, owing to the poor transfection efficacy and low immunogenicity. Nevertheless, DNA vaccines do offer certain advantages [25]. First, the use of plasmid DNA for vaccination is safer than certain traditional vaccines, in that it avoids the administration of a live virus. Second, plasmid DNAs tend to be more stable than proteins, viruses, or mRNAs, and they can be freeze-dried and maintained in long-term storage. Third, the production of these vaccines is more straightforward and cost-effective. In recent years, improved transfection methods, such as electroporation based on the use of electric pulses to perforate the cell membrane, have been developed in order to enhance plasmid transfer into cells. The use of adjuvants to boost the immune reaction has been further advance in the development of DNA vaccines, which has increased the suitability of DNA vaccines as an ideal type of vaccine for mass administration. In this context, the company Inovio performed one of the earliest vaccination studies targeting the MERS coronavirus in order to develop a new DNA vaccine for COVID-19 [26]. Immunization with the synthetic DNA-based vaccine (INO-4800) targeting the SARS-CoV-2 spike protein resulted in the strong expression of this protein, and it promoted antigen-specific T cell responses and the production of antibodies, which were able to bind to ACE receptors and neutralize SARS-CoV-2 infection [26]. Previously, Inovio had also developed similar DNA vaccines against the Ebola [27], SARS [28], MERS [29,30], and Zika [31] viruses. Other previous studies have similarly used DNA-based vaccines to generate immunity against *Toxoplasma gondii* in mice [24], and also to produce a T-cell-dependent antibody response to glutamic acid decarboxylase [23].

### 3.3. Viral Vector Vaccines

Although the use viral vectors for therapeutic purposes commenced in the late 1990s, the application of these vectors for disease treatment was primarily overshadowed by the death of Jesse Gelsinger, who was administered an adenoviral vector [32], as well as the development of leukemia in children with severe combined immunodeficiency (SCID) treated with retroviral vectors [33,34]. However, in recent years, significant progress in the development of viral vector vaccines has yielded encouraging results with respect to dendritic cells, and an increasing number of studies have begun to focus on the use of different viral vectors, including RNA (retroviral and lentiviral), adenoviral, and Adeno-associated virus (AAV) vectors [35,36,37,38]. Immunization based on viral vector vaccines entails cloning the immunogenicity-causing antigen in a pseudovirus, which lacks the ability to propagate and transfer in dendritic cells, thereby producing stronger immune stimulation than recombinant proteins [39].

#### 3.3.1. Retrovirus- and Lentivirus-Based Vectors

**Figure 1:**
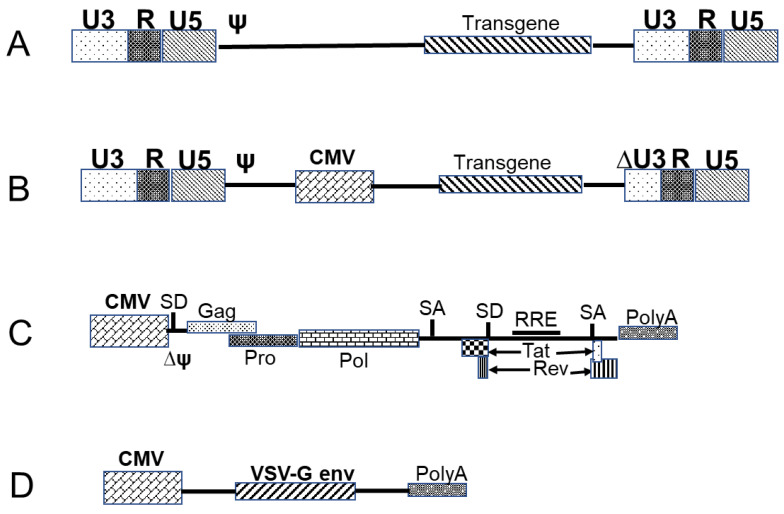
Retrovirus-based vectors. (**A**) Representation of a retrovirus-based vector, and (**B**) a SIN lentiviral-based vector. The long terminal repeat (LTR) is divided into three regions (U3, R and U5). The packaging of the viral RNA takes place via interaction between the packaging signal ψ and the viral proteins. The bonding of Rev to the rev response element (RRE) enables the transport of un-spliced or once-spliced RNA from the nucleus to the cytoplasm. (**C**) A helper plasmid with viral protein gag-pro-pol under the expression of the cauliflower mosaic virus (CMV) promoter. (**D**) A helper plasmid for the env protein.

Retroviruses have a single-stranded RNA genome that encodes all of the proteins that are required for replication, including structural proteins [40]. Of the studied retroviruses, Moloney murine leukemia virus (MMLV)-based vectors are amongst the most efficient engineered vectors with a high transduction efficiency in dividing cells, and they are characterized by good integration and high expression of transgenes [41]. Retroviral expression vector can carry genes of interest of up to 8 kb in size and a diverse range of envelope proteins can be used for packaging. These envelope proteins can be modified to recognize receptors that are only found on mouse and rat cells (ecotropic), or be amphoteric, thereby facilitating the targeting of a broad range of receptors on mammalian cells. Moreover, retroviral vectors can be pseudotyped with envelope proteins that are derived from other virus strains, such as vesicular stomatitis virus G-protein (VSV-G), which exhibits broad-spectrum tropism and facilitates the infection of non-mammalian cells [42]. Therapy using retroviral vectors has been demonstrated to be an efficient treatment for different disorders, including X-linked SCID (SCID-X1), chronic granulomatous disease (CGD), and adenosine deaminase-deficient SCID [43,44,45,46]. However, critical complications, such as leukemias in patients with SCID-X1 and myelodysplastic-like syndromes in patients with CGD [47], highlighted the limitations of the first generation of retroviral vectors. Subsequently, modified vectors with modest architectural changes and a more favorable profile than the first-generation retroviral vectors were developed [48,49,50,51,52,53]. These vectors are characterized by the deletion of the long terminal repeat (LTR) promoter region, which results in the self-inactivation (SIN) of the vector and good biosafety (Figure 1). A further type of retrovirus, lentiviruses, can infect non-dividing cells, such as dendritic cells and macrophages [54,55]. The packaging capacity of lentiviral vectors is similar to that of murine leukemia virus vectors, and they can be pseudotyped with different envelopes, including VSV-G [56]. Furthermore, lentiviral-based vectors exhibit strong and prolonged expression, owing to random chromosomal integration. However, this genomic integration has been associated with the development of leukemia in patients with SCID [33,34], which initially represented a considerable drawback for the use of retroviral vectors. Nevertheless, this disadvantage has recently been overcome by the development of new vectors that are characterized by targeted integration [57]. The development of non-integrating lentiviral vector (NILVs) [58], which have the capacity to express transgenes transiently in dividing cells or episomes in non-dividing cells and can also be introduced into different cell types, has been a further interesting advancement in the use of lentiviral vectors. In this regard, the Shenzhen Geno-immune Medical Institute has recently developed two lentiviral vector-based vaccines (Covid-19/aAPC and LV-SMENP-DC) for the treatment of COVID-19 infections [5]. These vectors have been constructed in order to harbor multiple viral genes as antigens, including conserved, structural, and protease protein domains, and the vaccines are currently undergoing phase 1 (Covid-19/aAPC) and phase 2 (LV-SMENP-DC) trials.

#### 3.3.2. Adenovirus-Based Vectors

**Figure 2:**
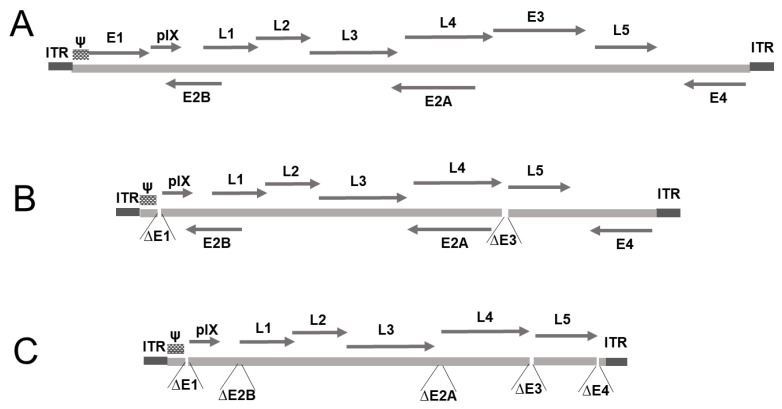
Adenovirus-based vectors. (**A**) A map of the human adenovirus type 5 (HAd5) genome. It consists of early genes (E1–E4) that suppress cell responses against the virus, and are responsible for the replication and regulation of viral transcription. The late genes (L1–L5) encode the structural proteins of the virus. (**B**) A first-generation adenovirus-based vector (8-kb packaging capacity) in which the E1 and E3 genes have been deleted. (**C**) A second-generation vector in which the E2 and E4 genes have been deleted to increase packaging capacity (14-kb packaging capacity). For packaging, the plasmid harboring the transgene is transfected with a helper plasmid for the expression of viral genes E1, E2, and E4.

Adenoviruses, which belong to the family *Adenoviridae*, are non-enveloped, double-stranded DNA viruses, approximately 90 nm in diameter. First discovered in 1953, they are known to infect humans and a range bird, reptile, fish, amphibian, and non-human primate species [59,60,61]. In humans, more than 100 types of adenovirus have been identified, some of which are implicated in respiratory infections, conjunctivitis, or gastroenteritis [59,62]. The linear double-stranded DNA of the virus measures between 25 and 48 kb and it includes non-coding inverted terminal repeats (ITRs) at both ends and genes encoding approximately 35 proteins (Figure 2A) that are expressed in two different phases, i.e., early genes, including E1A, E1B, and E2–E4, and five late genes (L1–L5). The early genes play important roles in gene regulation in the host cell and in the initiation of virus replication, whereas the late genes encode structural proteins that are essential for capsid assembly [62]. Of the early genes, the E3 gene is not essential for adenovirus replication, whereas, E1, E2, and E4, are necessary. In the first-generation adenovirus-based vectors, which had a packaging capacity of approximately 8 kb, the E1 and E3 genes were deleted (Figure 2B), whereas, in the second-generation vectors, with 14-kb packaging capacity, the E2 and E4 genes were deleted (Figure 2C), and, in the case of high-capacity adenovirus-based vectors, all of the genes were deleted, leaving only the *cis*-acting sequences necessary for viral DNA replication and packaging, which enabled the packaging of transgenes of up to 35 kb in size. The genes that are required for replication can be stably expressed in packaging cells or cloned into helper plasmids and co-transfected with the transgene plasmid [63]. In previous studies, the most commonly used adenoviral vector has been based on human adenovirus serotype 5 (AdHu5), which has been found to be efficient in inducing immune responses in preclinical and clinical studies, as well as in gene therapy applications [64,65,66,67]. Recently, an adenovirus-based vector has been used in order to develop a new COVID-19 vaccine [5]. The use of adenovirus-based vectors has several advantages, including strong transgene expression and immune responses via an induction of innate immunity, allowing for large-scale production and purification, and ensuring safe human application. Furthermore, they can be delivered via mucosal or systemic routes [68,69,70]. Collectively, these advantages have contributed to establishing adenovirus-based vectors as among the most successful strategies in medical research.

#### 3.3.3. Adeno-Associated Virus Vectors as a Platform for Vaccination

**Figure 3:**
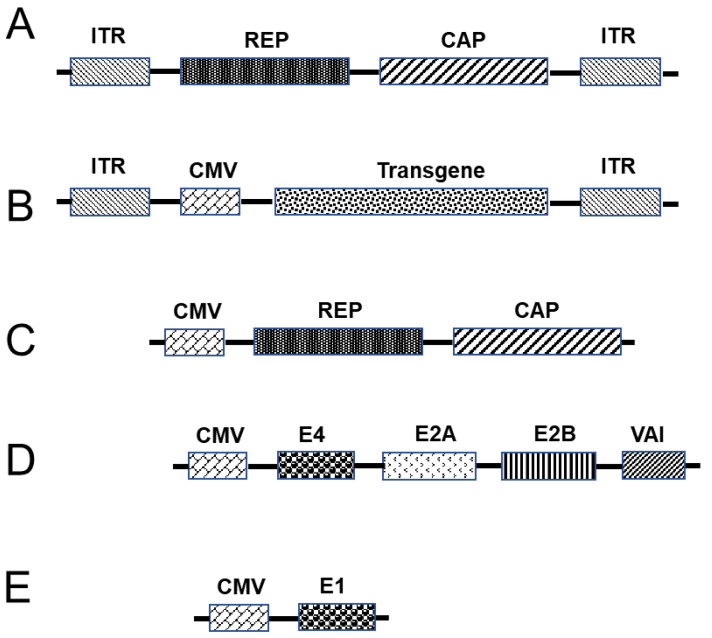
Adeno-associated-based vaccine. (**A**) The wild-type adeno-associated virus (AAV) genome can be modified by replacing the gene for replication (REP) and structural genes (CAP) with the transgene of interest. (**B**) A transgene containing promoter and regulatory elements is cloned between the two inverted terminal repeats (ITRs) to generate a recombinant AAV (rAAV) genome. For the production of rAAV particles, the construct carrying the transgene should be co-transfected in permissive cells with plasmids that contain REP and CAP genes (**C**), and adenovirus helper genes (**D**,**E**).

Adeno-associated viruses (AAVs) are small non-enveloped viruses that belong to the genus *Dependovirus* within the family *Parvoviridae* [71,72,73]. The first AAV was discovered in 1965 [72] and, during the subsequent 20 years of research, several important aspects of AAV were characterized, including the genome structure [74,75,76,77], infection latency [78,79,80,81], replication/transcription [82,83,84], virion assembly [85], genetic characteristics [86,87], and sequence of the entire genome [88]. The virion of the AAV is a single-stranded particle of approximately 4.7 kb in length that can either be a sense or anti-sense strand [89] encapsulated within a 25-nm capsid [71]. The AAV genome includes an ITR at either end, which serve as origins of replication and packaging signals. With respect to replication, the genome contains a single replication (rep) gene encoding four proteins (Rep4, Rep52, Rep68, and Rep78), a capsid (cap) gene encoding three subunits via differential splicing and translation variants, and an assembly activating protein (AAP) that is localized within the capsid sequence that promotes virion assembly [90,91]. In response to an interaction between the capsid and a cell receptor, the AAV particles undergo a series of pH-dependent structural modifications within endosomes [92] and subsequently enter the nucleus by way of interacting with the nuclear pore complex following endosomal escape [93,94,95], during which the single-stranded DNA genome is released. A second strand is synthesized from the self-primed ITR at the 3′-end [96,97], followed by strand annealing via base pairing. The double-stranded genome thus generated undergoes circularization via intra- or intermolecular genome recombination at the ITRs [98], which stabilizes the recombinant AAV (rAAV) genome as episomal DNA, leading to persistent gene expression in post-mitotic cells. Although AAVs lack the capacity to autonomously replicate, they can infect both non-dividing and dividing cells in different hosts, including humans and non-human primates [71]. However, in order to reproduce in cells, these viruses require the mediation of a helper virus, such as herpes simplex virus (HSVs) or an adenovirus [99]. Although AAV infection is common in humans, these viruses are not known to cause any disease [100,101,102], and, consequently, AAVs are considered ideal vectors for gene transfer and vaccination [103,104]. Nevertheless, the packaging capacity of these viruses is limited and, thus, to maximize loading, the entire genome, with the exception of the ITRs, must be removed (Figure 3), leading to low cytotoxicity and immunogenicity when AAVs are delivered in vivo. Since its introduction in the 1980s, rAAVs have become the gold standard for gene transfer [105]. In terms of packaging, the transgene is cloned into a plasmid between the ITRs and transfected into cells along with helper plasmids (Figure 3), namely, one for the rep and cap genes and another, including adenovirus helper genes [106,107]. This strategy facilitates relatively straightforward packaging of the gene of interest [108,109]. Different studies have confirmed the efficacy and safety of rAAVs with respect to delivering genes in target cells during several preclinical and clinical trials for the treatment of genetic diseases, including hemophilia, spinal muscular atrophy, inherited retinal disease, and lipoprotein lipase deficiency [110,111,112], and they have been licensed for treatment [113,114]. Given the therapeutic promise of these vectors, recent studies have focused on developing an AAV-type COVID-19 vaccine [5].

### 3.4. RNA-Based Vaccines and Nanoparticle (NP) Formulations

RNA-based vaccines are the most recent development in the quest to produce safe and efficacious means of vaccination. One of the major factors that has hitherto prohibited the use of RNA for vaccination is its low stability. Furthermore, RNA only enables transient expression and it is negatively charged and, consequently, the use of additional substrates is necessary for facilitating the entry of RNA into cells. However, recently, different strategies have been developed to enhance mRNA stability and the delivery of RNA into cells, which have contributed to making RNA-based strategies among the most efficient methods of vaccination.

#### 3.4.1. RNA-Based Vaccines

**Figure 4:**
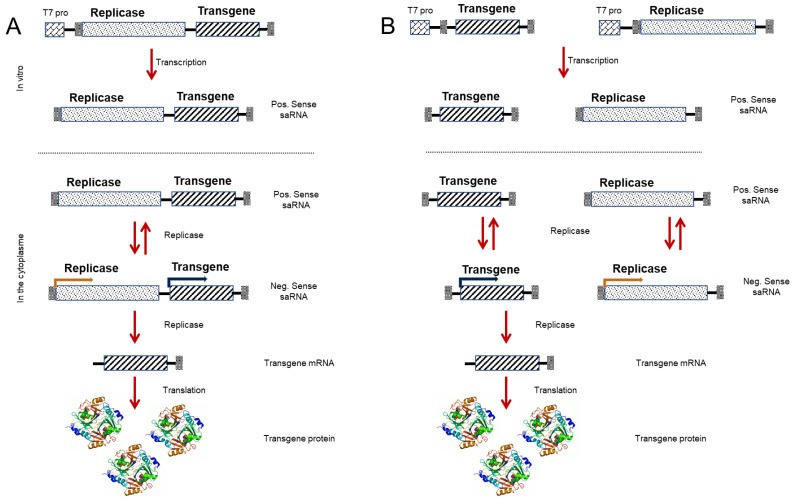
RNA based vaccines. Plasmid DNA carrying replicase genes (for the replication of RNA) and/or the transgene (which encodes the gene of interest) can be transcribed in vitro using a T7 promoter transcription system to generate replicons or positive sense RNAs (Pos sense RNA). (**A**) The replicon RNA encodes the replicase machinery and the transgene are delivered into the cell using lipofectamine or similar synthetic formulations. Within the cytoplasm, the replicon RNA self-replicates and produce transgene mRNA from the subgenomic promoter, which is translated to protein. (**B**) The replicon RNA encoding the replicase machinery and the transgene are delivered “in *trans*”. Within the cytoplasm, the replicon RNA self-replicates and produces transgene mRNA using a subgenomic promoter, which is subsequently translated to protein.

mRNA-based vaccines have already been used in the treatment of different diseases [115,116,117]. However, being negatively charged, the efficiency of mRNA transfection tends to be very low, thus necessitating the use of other substrates in order to facilitate the delivery of mRNA into cells, among which lipid nanoparticles (LNPs) are some of the most widely used transport vehicles [118,119]. Although the mechanisms whereby mRNAs are delivered by LNPs are incompletely understood, it has been established that mRNA-LNP complexes are taken up by endocytosis after interaction with the cell membrane [120], and are thereafter routed to the endosomes. As a consequence of a change in pH, the residual amines of the LNPs subsequently disrupt the endosome membrane, leading to the endosomal escape of mRNA into the cytoplasm, which, in turn, enables the transient expression of mRNA in order to produce a particular protein [121]. Given that entry into the cell nucleus is not a prerequisite for mRNA vaccine antigen expression, the expression is only transient and, accordingly, the risk of integration into the host DNA is negligible, which is one of the salient advantages of these vaccines. However, this transient expression of mRNA is typically low, due to rapid degradation by cytoplasmically localized endonucleases. In an effort overcome these limitations, several recent studies have investigated strategies to increase mRNA stability, thereby facilitating high protein production. These strategies include the use of nanoparticle (NP) delivery methods [122] and modified nucleosides [123], which have been reported to confer enhanced mRNA stability and improved bioavailability for the production of larger quantities of antigens. Interestingly, in this regard, Beissert et al. recently identified a new generation of RNA molecule with vaccine potential, referred to as self-amplifying RNA (saRNA) [124]. saRNAs are based on the genome of alphaviruses, in which the RNA replication genes remain intact, whereas the structural genes are deleted. The delivery of saRNAs can be achieved while using plasmid DNA, the transcription of which is dependent on prior entry into the nucleus. In addition, the RNA can be transcribed in vitro and transported to the cytoplasm while using viral vectors or non-viral NPs (Figure 4), thereby facilitating the expression of antigens in both non-dividing and dividing cells, as well as promoting a more extensive and stronger immune response than mRNA [125]. A further development in this context is a strategy that is based on the delivery of two constructs in trans-amplifying RNA (taRNA), one of which carries the transgene of interest and the other harbors a replicase gene [124]. In response to immunization with nanogram doses of the antigen, the authors noted high antibody production in the treated mice [124]. This approach tends to have a better safety profile than normal saRNA vaccines, which could be attributed to the use of two different RNA constructs that further reduces the possibility of engineered viral particles being transferred into host cells. The first COVID-19 vaccine to enter clinical trials was an RNA-based vaccine and it has been anticipated to make a considerable contribution in the fight against the current pandemic [126]. In addition, the application of as little as 50 ng of taRNA in mice was found to result in the production of antibodies against influenza hemagglutinin antigen [124]. Furthermore, saRNA viral vectors have been used for the expression of tumor and viral antigens, which results in strong cellular and humoral immune responses, and such vaccines have yielded promising results in clinical trials that were conducted to assess efficacy against the Ebola virus. RNA-based vaccines provide a potentially rapid and straightforward platform for vaccination and, depending on the sequence of the gene of interest, these vaccines can be produced within a few weeks, with clinical trials being initiated within a couple of months. In addition, the possibility of using different NP strategies to facilitate RNA transfer within cells offer considerable scope for broadening the therapeutic approaches. Collectively, these factors contribute to making RNA-based vaccines potentially the most promising strategy available for meeting the urgent demand for an effective COVID-19 vaccine.

#### 3.4.2. NP Formulation for RNA Delivery

For more than two decades, researchers have been attempting to address the considerable challenges related to the therapeutic application of RNAs, notably intracellular distribution, stability, and stimulation of an immune response. In this regard, the priority is to develop procedures for the effective and nontoxic delivery of curatively applicable RNAs, the lack thereof has, to date, limited their use in humans. Efficient distribution mechanisms are urgently required, and in this respect, several nanomaterials have recently been engineered to deliver RNA, protect mRNA against extracellular degradation, and promote endosomal leakage subsequent to cellular uptake. NP networks have considerably broadened the scope of RNA-based therapies and, thereby, provided a basis for prospective applications in protein substitute therapy, therapeutic vaccinations, cancer immunotherapy, and gene editing [122]. NPs, including liposomes and polymeric, dendrimer, and metal NPs, such as silver, gold, and quantum dots, can be used as carriers to conjugate or encapsulate siRNA for gene silencing [127], and polyethylenimine has been widely used for the delivery of siRNA in vitro and in vivo via electrostatic interactions [128,129].

The concept of using exogenous RNA for protein expression dates back to 1978, when Dimitriadis demonstrated that rabbit globin mRNA entrapped in liposomes could be incorporated into lymphocytes [130]. However, as mentioned previously, RNA has yet to be used as a therapeutic agent, owing to limitations, such as unsatisfactory stability and cell penetration and high development costs [122]. Nevertheless, circumstances have gradually improved over the past few years, as our knowledge regarding nucleic acid chemistry has increased and the manufacturing costs for RNAs have declined [131]. As transport vehicles, NPs protect RNA against degradation by RNases, enhance cellular absorption, and promote endosomal escape, with the subsequent cytosolic expression of functional proteins [122].

As an example of the efficacy of NP-mediated RNA delivery, Pardi et al. engendered mRNA-LNPs, which were injected into mice at doses of between 0.005 and 0.250 mg/kg via six different routes, and accordingly detected high levels of protein translation that could be measured by in vivo imaging [132]. mRNA that was administered via subcutaneous, intramuscular, and intradermal injection was found to be translated locally at the site of injection for up to 10 days, and high levels of protein production could be obtained in the lungs following intratracheal administration of mRNA. Further, intraperitoneal and intramuscular administration was found to contribute to the systematic transfer of mRNA-LNPs into the liver for 1–4 days. Thus, these results indicate that LNPs can be transported by passive targeting [132]. The potential efficacy of RNA therapy has been investigated with respect to various genetic disorders, and lipid-derived nanomaterials are considered to be among the most promising biomaterials for effective RNA delivery [133]. A longstanding challenge has been to develop safe and effective delivery mechanisms for therapeutic biomacromolecules [134] and, in this regard, Sedic et al. [135] published a safety review of LNPs that were loaded with human erythropoietin mRNA delivered to both Sprague–Dawley rats and cynomolgus monkeys, and defined their pharmacology, pharmacokinetics, and safety profiles. The authors found that. following the intravenous application of mRNA-LNPs to rats and monkeys, plasma concentrations reached their highest levels at 6 h after administration.

The administered RNA-NP preparations undergo complex or multistage cellular absorption (endocytosis) via multiple mechanisms, including clathrin-dependent and clathrin-associated pathways, whereby they become enclosed in membrane-bound organelles, referred to as endosomes [136], from which they must subsequently be released into the cytosol, via endosome–lysosome formation, in order to enable mRNA translation. Lysosomes regulate mTOR signaling, as well as cell proliferation and mRNA translation. By activating mTOR, the formation of endosome–lysosome complexes can either enhance or suppress the mRNA delivery pathway [137]. Although the mechanisms that are associated with endosomal escape have yet to sufficiently determined [138], it has been proposed that the release of nucleic acids from endosomes is spatiotemporally limited. Given the comparatively low levels of intact introduced mRNA, it is believed that most undergo lysosome-mediated degradation [139]. NPs are assumed to counter the influence of mild to moderate acidosis by increasing endosome osmotic pressure via endo-lysosomal maturation. A further hypothesis maintains that cationic NPs associate with anionic lipids on the endosomal membrane in order to produce a hexagonal phase. [137]. Additionally, membrane transporters can facilitate the efflux of nucleic acids from endosomes, an example of which is the transmembrane cholesterol transporter that is found on late endosomes/lysosomes that mediates the efflux of siRNA to the extracellular milieu [140]. Moreover, it has been established that antisense oligonucleotides can interact with cellular proteins to facilitate transport to the cell membrane [141]. However, despite recent advances in our understanding of endosomal escape routes, this is an area warranting further in-depth study [138].

The use of liposomes to transfer genes has multiple applications in a number biomedical fields. These vesicles have been shown to contribute to stabilizing therapeutic drugs and genes, resolving cell and tissue absorption barriers, and enhancing the bio-distribution of compounds to in vivo target sites [142]. In this regard, Pisal et al. described the use of lipid-based transport vehicles that are characterized by diverse molecular architectures, including liposomes, solid-lipid NPs, oily suspensions, submicron lipid emulsions, lipid implants, lipid microbubbles, inverse lipid micelles, cochlear liposomes, lipid microtubules, and lipid microcylinders [143], whereas other studies have demonstrated the successful delivery of mRNA and translation of DNA in different cells while using such vehicles [144,145].

Polymeric NPs are a further class of nanostructure distribution networks, of which several types have been synthesized and characterized, including polyamines, polypeptides, and triblock polymers. Polyethyleneimine (PEI) is a further example, which is used as a cationic polymer for the delivery of nucleic acids. PEI polymers comprise linear or branched chains that can readily attract and carry nucleic acids and have a proton sponge effect that can facilitate endosomal escape [122]. PEI dendrimers can also be employed for the delivery of mRNA. Moreover, mRNA delivered via polymeric NPs has been demonstrated to promote the development of good immunity against influenza H1N1, Ebola, *Toxoplasma gondii*, and Zika [146].

Protamine is an FDA-approved arginine-rich protein that is used as an insulin transport system, and several studies have shown that lipid/polymer protamine/RNA complexes can be used in order to enhance mRNA stability and tumor aggregation. While sing different portions of a cationic lipid (1,2-dioleoyl-3-trimethylammonium-propane) or the cationic biopolymer protamine as models, it has been demonstrated that NPs comprising a mixture of lipidic and polymeric materials can function as carriers for mRNA transfection. The results indicated that both hybrid structures incorporating lipid and polymer facilitated substantially higher stable transfection than lipid/mRNA and polymer/mRNA particles alone [147]. Researchers [148] have also developed protamine-RNA LPRs (loaded liposomes) targeting herpes simplex virus 1-thymidine kinase. These LPRs have be shown to inhibit tumor development in a human lung cancer xenograft rat model. Subsequently, poly(π-caprolactone) was used in order to develop protamine/RNA complexes as pH-sensitive NPs, and these core-shell NPs as pH-dependent formulations were observed to produce RNA in three cell lines [149].

Metal-based NPs, such as nano-gold and nano-silver, and nano-metal oxides (zinc oxide, titanium dioxide, iron oxide, and quantum dots) can be used for biological and medical applications [150]. Gold and silver NPs are considered to be particularly important and applicable tools in nanotechnology. Gold NPs (AuNPs), polymer-lipid hybrid NPs, and peptide complexes can be used to deliver mRNA, whereas thiolated AgNPs, loaded or coated with a short DNA oligonucleotide, have been demonstrated to undergo complementary binding with unique RNA sequences [151]. AuNPs are considered to be an appropriate platform for the delivery of nucleic acids and, in this regard, it has been observed that AuNP/RNA complexes not only cause cell apoptosis in vitro, but also inhibit xenograft tumor development in mice following subcutaneous injection [152]. AuNPs can be synthesized as uniform materials with low size dispersity and they are readily functionalized via modification with different multifunctional monolayers, moieties, and targeting agents. Moreover, their toxicity and biodistribution in vivo can be controlled by optimizing particle size and surface functionality, and it has been demonstrated that these NPs can be readily engulfed by reticule endothelial cells [153,154,155]. As an example of the therapeutic potential of AuNPs, Yeom et al. [152] injected AuNPs that were coated with an mRNA encoding Bcl-2-associated X (BAX) protein, a pro-apoptotic factor, into mice xenograft tumors and observed the subsequent release of mRNA and production BAX protein, resulting in the inhibition of tumor growth.

### 3.5. Vaccine Adjuvants

Vaccines have been extensively established as powerful tools in combating diverse diseases. Traditional vaccines, including the use of inactivated pathogens or pathogens with reduced virulence, are characterized by the induction of strong immunogenicity, low production costs, and relatively straightforward preparation processes. However, generally, they tend to have poor safety profiles [11], which has led to the emergence of alternative next-generation vaccines, including recombinant protein vaccines, DNA-, virus-, and RNA-based vaccines with better safety profiles. However, these novel vaccines, particularly those employing RNA, plasmids, and recombinant proteins, are typically characterized by low immunogenicity [21]. Consequently, there is an urgent need to develop adjuvants that can be used in order to enhance the immune reaction and increase vaccine efficacy. Adjuvants enhance antigen presentation in antigen-presenting cells (APCs), thereby improving immunogenicity and ensuring long-term protection. As long ago as 1930, aluminum adjuvants were first used in clinical trials and they are still used in approximately 80% of those vaccines delivered in adjuvants [156]. Aluminum adjuvants can stimulate the immune system via different pathways, and they have been shown to bind to and alter the membrane structure of dendritic cells [157]. Moreover, they may either induce apoptosis or stimulate NLRP3 inflammasomes in order to produce threat signals, thereby initiating an immune reaction [158,159]. However, as the use of aluminum adjuvants can be associated with the induction of weak cellular immunity and they are ineffective against intracellular viral infection [160], a new type of adjuvant containing monophosphoryl lipid A and aluminum hydroxide has been developed for vaccines for hepatitis B and papillomaviruses [161]. Similarly, a combination of aluminum and CpG has been used against malaria [162], and nano-aluminum adjuvants [163] have also been employed. Furthermore, Jiang et al. developed PEG-coated nano-aluminum particles that could enter lymph nodes and showed synergistic effects with CpG [164]. Recently, different companies have developed emulsion adjuvants, being classified as oil-in-water emulsion adjuvants, including AF03, MF59, AS02, and AS03 [165,166,167], or water-in-oil emulsions, including Montanide ISA51 and ISA720 [168,169]. These emulsion adjuvants can be used to induce high humoral immunity via different interactions. For example, in the case of MF59, this effect is attributable to the induction of threat signal release from muscle cells at the injection site. Furthermore, the effect was found to be associated with apoptosis-related speck-like proteins (ASC) containing a caspase recruitment domain, and the activation of the MyD88 gene [170]. More recently, Xia et al. coated a core comprising a mixture of squalene and all-*trans* retinoic acid with a shell of poly(lactic-*co*-glycolic acid), which was found to enhance the expression of CCR9 on the surface of dendritic cells, resulting in antigen uptake, homing of these cells in the lymph nodes, and, consequently, the induction of strong mucosal immunity [171].

AS01, which is used as an adjuvant with vaccines for herpes zoster and malaria, is an adjuvant system of particular interest. This preparation is based on liposomes that are derived from cholesterol in combination with dioleoylphosphatidyl-choline and two immunostimulants, namely, QS21 (purified saponin) and MPL (a derivative of lipopolysaccharide), which have a synergistic effect [172,173]. Although QS21 is potentially toxic, cholesterol reduces this toxicity, thereby improving the safety of the adjuvant. After administration, QS21 translocates to the lymph nodes, wherein it accumulates and stimulates caspase-1, which is followed by the production of high-mobility group protein B1 and activation of the TLR4-MyD88-related pathway [174]. A further adjuvant derived from AS01 is AS015, which, combined with CpG oligodeoxynucleotide 7909, has been used in conjunction with a vaccine for melanoma [175,176], and it can also enhance anti-cancer activity [177,178]. Other researchers have used [poly(lactic-*co*-glycolic acid)] or natural chitosan, which have good safety and biocompatibility profiles, to protect antigens and enhance antigen uptake by APCs [171,179]. Chitosan adjuvants comprise particles of differing forms, sizes, pH values, and surface charges. In the case of acid-soluble chitosan adjuvants, following uptake by APCs, the particles are solubilized in lysosomes, thereby promoting changes in lysosome pH and conformation and, consequently, the release and expression of the antigen. Subsequent to degradation, APCs present the antigen to naïve T cells, which are accordingly activated [179].

Protein adjuvants are the final types of adjuvant described in this review, which include heat shock protein (HSP), GM-CSF, flagellin, and cytokine (e.g., IL2)-based preparations. Protein adjuvants are delivered and expressed as a single protein in combination with the antigen and they are characterized by a good safety profile. Moreover, the findings of previous studies have indicated that co-delivery of the antigen with these adjuvants can significantly strengthen the immune reaction [180,181,182].

## 4. Conclusions

The use of vaccines can be traced back to the 18th century, when diseases, such as smallpox, were successfully treated while using pathogens with reduced virulence. Since that time, vaccination strategies have undergone a continual evolution and a number of different vaccine types have been used to treat diseases that are caused by a diverse range of pathogens, as well as in combatting cancer. In addition to the more traditional methods of vaccination, there is an ongoing emergence of new-generation technologies, including viral vector-based techniques and RNA-based vaccines. Progress in the development of each of these novel vaccine types has had to contend with multiple challenges, not only with respect to the underlying scientific concepts, but also in terms of the logistics of mass production, distribution, storage, and mass vaccination. During the development of vaccines for the treatment of Covid-19, the efficacy of all strategies developed thus far has been assessed. On the basis of present evidence, it can be concluded that the RNA-based vaccines are probably superior with respect the timescale of development; however, the associated costs tend to be higher than those of other strategies, due to the necessary specifications of production, distribution, and storage.

## Data Availability

No new data were created or analyzed in this study. Data sharing is not applicable to this article.
